# Chitosan Hydrogel Beads Supported with Ceria for Boron Removal

**DOI:** 10.3390/ijms20071567

**Published:** 2019-03-28

**Authors:** Joanna Kluczka, Gabriela Dudek, Alicja Kazek-Kęsik, Małgorzata Gnus

**Affiliations:** 1 Department of Inorganic, Analytical Chemistry and Electrochemistry, Faculty of Chemistry, Silesian University of Technology, B. Krzywoustego 6, 44-100 Gliwice, Poland; Alicja.Kazek-Kesik@polsl.pl; 2 Department of Physical Chemistry and Technology of Polymers, Faculty of Chemistry, Silesian University of Technology, ks. M. Strzody 9, 44-100 Gliwice, Poland; Gabriela.Maria.Dudek@polsl.pl (G.D.); Malgorzata.Gnus@polsl.pl (M.G.)

**Keywords:** biopolymer, cerium dioxide hydrate, boron adsorption

## Abstract

In this study, a chitosan hydrogel supported with ceria (labelled Ce-CTS) was prepared by an encapsulation technique and used for the efficient removal of excess B(III) from aqueous solutions. The functionalisation of chitosan with Ce(IV) and the improvement in the adsorptive behaviour of the hydrogel were determined by SEM-EDS, FTIR, XRD, and inductively coupled plasma optical emission spectrometer (ICP-OES) analyses and discussed. The results demonstrate that Ce-CTS removes boric acid from aqueous solutions more efficiently than either cerium dioxide hydrate or raw chitosan beads, the precursors of the Ce-CTS biosorbent. The maximum adsorption capacity of 13.5 ± 0.9 mg/g was achieved at pH 7 after 24 h. The equilibrium data of boron adsorption on Ce-CTS fitted the Freundlich isotherm model, while the kinetic data followed the Elovich pseudo-second-order model, which indicated that the process was non-homogeneous. The dominant mechanism of removal was the reaction between boric acid molecules and hydroxyl groups bound to the ceria chelated by chitosan active centres. Due to its high efficiency in removing boron, good regeneration capacity and convenient form, Ce-CTS may be considered a promising biosorbent in water purification.

## 1. Introduction

The concentration of boron in water is regulated in various countries throughout the world. In Poland and the rest of the European Union, the recommended level of boron in drinking and irrigation water and in wastewater discarded to the environment is 1.0 mg/dm^3^. This is because boron—a significant micronutrient for plants [1]—is harmful in excess. In humans, a high content of boron may lead to damage to the nervous system [2].

Various techniques have been used in the removal of boron species from water and wastewater, including adsorption, ion exchange, co-precipitation, ultrafiltration, reverse osmosis and electrodialysis. Among these techniques, adsorption has been found to be a superior technique due to its ease of design, operational simplicity and good efficiency with regard to the removal, not only of boron, but also other pollutants [3,4,5,6,7]. In the last two decades, numerous papers have presented results for the adsorption of B(III) using various materials, such as active carbons [8,9,10], zeolites [11,12], metal oxides [13,14], nanoparticles [15,16] and waste materials [17,18]. 

Chitosan (CTS), which contains functional hydroxyl (OH) and amino (NH_2_) groups, is one of the most abundant biopolymers. It is extracted by a deacetylation procedure from chitin. This polymer has shown a high adsorption potential for various aquatic pollutants in water treatment [19]. Various metal cations, oils and dyes are easily attracted by these groups, due to the chelating effect and electrostatic attraction [20,21,22]. Some typical anions, such as fluoride, nitrate and phosphate, may undergo fixation by electrostatic attraction or ion exchange in acidic media on protonated amine functions [23,24,25]. The electrostatic attraction mechanism has also been reported for the binding of a series of metals that form anionic complexes in acidic solutions, e.g., Cr(VI) and Mo(VI) [22]. Unfortunately, the adsorption of boron species onto chitosan is characterised by the biopolymer not having a high enough capacity [26]. This is because, in a low pH solution, B(III) mainly exists in the inert form of B(OH)_3_. Under such conditions, electrostatic attraction does not occur. At a higher pH, when borate anions are the dominant forms in the solution, chitosan is not protonated to yield ammonium ions (NH_3_^+^), and there is instead a repulsion between the negatively charged surface of chitosan and B(OH)_4_^−^ ions. Therefore, many ways of functionalising chitosan in order to obtain greater applications have been reported [27]. Among them, rare earth metal-binding chitosan composites have attracted researchers’ attention because of their active properties and high adsorption capacity [21,28,29,30]. 

Cerium dioxide is one of the most abundant and cheapest rare earth oxides, possessing resistance to acids and bases and not eluting during the removal of harmful substances in water [29]. Cerium has a good chelating ability with the functional groups present in chitosan and can possess a high adsorption capacity because of its positive charge [21,30]. Biopolymers based on cerium(III) immobilised cross-linked chitosan (CTS-Ce) composites have been prepared and employed for the removal of fluoride and phosphate from water [28,29,31,32]. Additionally, a fibrous mat of chitosan/polyvinyl alcohol, containing cerium(III), was used for the removal of chromium(VI) from aqueous solutions [33].

Until now, boron has not been removed on such adsorbents based on chitosan and cerium ions, e.g., CTS-Ce, CTS/PVA-Ce, etc. Moreover, Ce^4+^ ions have never been previously used to modify chitosan; only Ce^3+^ salts have been used as an additive to a carrier in the above-cited papers. Therefore, a strategy of fabricating composites by immobilising cerium(IV) ions and chitosan is a novel and very promising concept. The objectives of this study are the following: (i) to synthesise the new composite (Ce-CTS) via a co-precipitation and coagulation method and to characterise its structure before and after adsorption using a Fourier-transform infrared (FTIR) spectrometer, scanning electron microscope (SEM) and X-ray diffractometer (XRD); (ii) to study the adsorption isotherms of B(III) on Ce-CTS composites in batch mode; (iii) to study the adsorption of boron on cerium dioxide hydrate; and (iv) to discuss the possible reasons and mechanisms for the adsorption of B on Ce-CTS composites. 

## 2. Results and Discussions

### 2.1. Ce-CTS Composite Characteristics

The prepared composite of chitosan supported with Ce(IV) was in the form of light yellow– beige hydrogel beads with an average diameter of about 4 mm (Figure S1 in the Supplementary Materials), which, after drying and grinding, became a powder (<0.45 mm) composite and remained a pale yellow–beige colour. 

**Figure 1:**
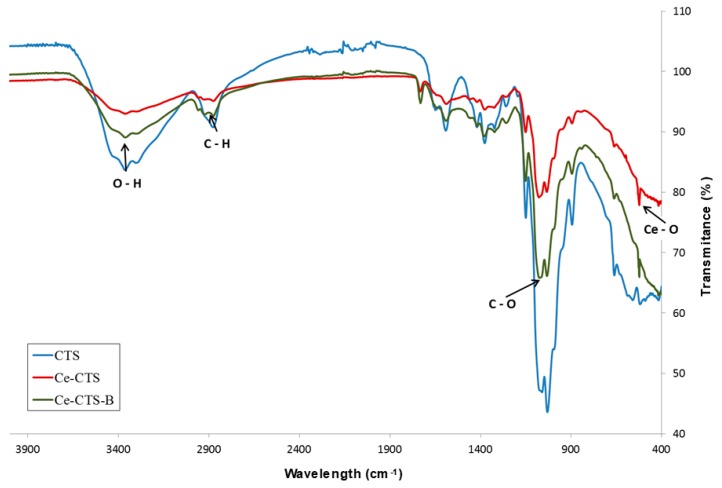
The FTIR spectra of the unmodified chitosan (CTS) and the chitosan composite supported with Ce(IV) before (Ce-CTS) and after (Ce-CTS-B) boron adsorption.

Figure 1 shows the FTIR spectra of the unmodified chitosan (CTS) and the prepared chitosan composite beads filled with Ce(IV) particles (Ce-CTS). Characteristic peaks for chitosan show the amide (C=O) stretch at 1651 cm^−1^, the C–N stretch at 1594 cm^−1^, the bending due to N–H stretching and the absorptions due to C–H stretching at 2890 cm^−1^ and the C–H bending at 1381 cm^−1^. C–O–C antisymmetric stretching can be seen at 1152 cm^−1^, and the C–O skeletal stretch characteristic of polysaccharides is observed at 1034 cm^−1^. The inorganic filler, i.e., cerium dioxide particles, shows absorption bands at 523 cm^−1^ [34]. After adsorption, in the spectrum of the Ce-CTS-B beads, more intensive bands than for Ce-CTS can be observed at 3400 cm^−1^, 2900 cm^−1^ and 1069 cm^−1^, due to the interaction of the OH groups of CTS and Ce with boron.

**Figure 2:**
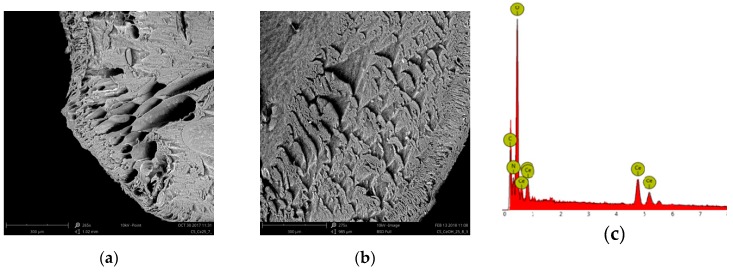
SEM images of the cross-section of the chitosan composite supported with Ce(IV): (**a**) Ce-CTS before adsorption; (**b**) Ce-CTS-B after boron adsorption; and (**c**) the EDS spectrum of Ce-CTS.

The SEM images of the Ce-CTS composite before and after adsorption of boron are shown in Figure 2. Comparing the Ce-CTS and Ce-CTS-B beads, more open structures are observed in the Ce-CTS bead before boron adsorption. In addition, the SEM image shows a well-ordered honeycomb structure with pores in each layer. The large surface area provided by the porous structure of Ce-CTS beads exposes more available active sites during the adsorption process for the capture of boron. After adsorption, such open pores are not visible, and the whole structure becomes more compact and closed. The EDS measurement was carried out to confirm the elements in the composite. The EDS spectrum of the Ce-CTS composite shows the presence of C, N, O and Ce in the investigated sample, confirming the incorporation of Ce^4+^ ions into the chitosan matrix.

**Figure 3:**
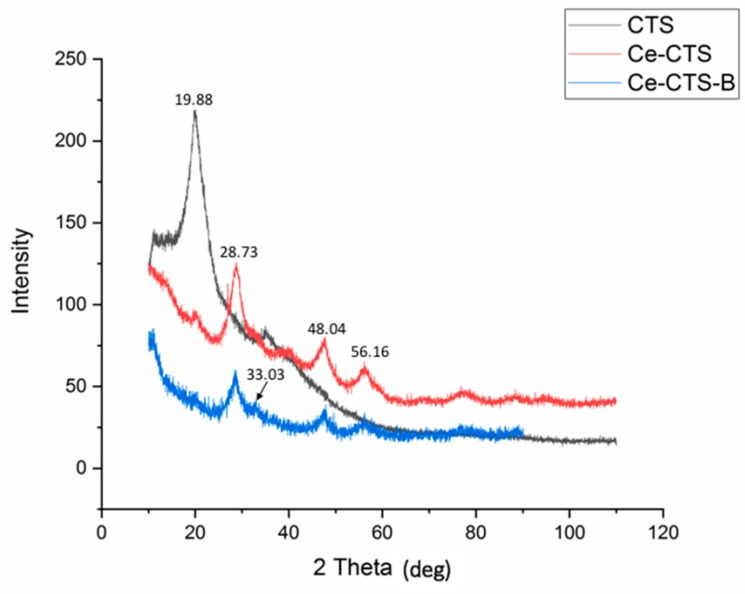
The XRD spectra of the unmodified chitosan (CTS) and the chitosan composite supported with Ce(IV) before boron adsorption (Ce-CTS) and after boron adsorption (Ce-CTS-B).

Figure 3 presents the XRD pattern of the unmodified CTS and the Ce-CTS composite before and after boron adsorption. The addition of cerium caused the chitosan to be enriched with the cerium-based compound. The particles of cerium were not well crystallised, or the nanoparticles had a very small size; thus, the identification of all of the peaks was not possible. However, on the XRD pattern, some of the broad peaks were registered, and their positions were at 28.73°, 33.03°, 48.04°, 56.16° and the broad bump between 74.53–82.23° 2Theta. The position of the peaks might indicate that ceramic oxide particles, CeO_2_, were formed (JCPDS card #89-8436). Similar results were presented in [35], where chitosan was modified by cerium compounds to obtain a bio-nanomaterial. The majority of the powder sample consisted of chitosan, which usually exhibits an amorphous phase; however, characteristic broad signals between 18 and 22° 2Theta, and a small 37–39° 2Theta signal were registered. No differences in the XRD patterns were observed between the Ce-CTS and Ce-CTS-B samples.

The content of cerium in the Ce-CTS composite hydrogel beads (after dissolving three dry samples in concentrated HNO_3_) was equal to 1.71 ± 0.20%. This confirmed once again the presence of cerium in the new composite material.

### 2.2. The Possible Mechanism of Ce-CTS Composite Formation

Cerium is the only lanthanide that forms stable solids in the IV oxidation state. In an aqueous solution, it is in the form of a hydrate [Ce(H_2_O)_n_]^4+^ (*n* >6). In this study, cerium(IV) nitrate was used as a precursor of the Ce-CTS composite. Ce(NO_3_)_4_ is a substance that is soluble in water but undergoes hydrolysis and then precipitation according to the following equations [36]:

[Ce(H_2_O)_n_]^4+^ + 4 H_2_O → [Ce(OH)_4_(H_2_O)_n−4_] + 4 H_3_O^+^ (1)

[Ce(OH)_4_(H_2_O)_n−4_] + 4 H_3_O^+^ → CeO_2_·nH_2_O + 4 H_2_O (2)

**Scheme 1:**
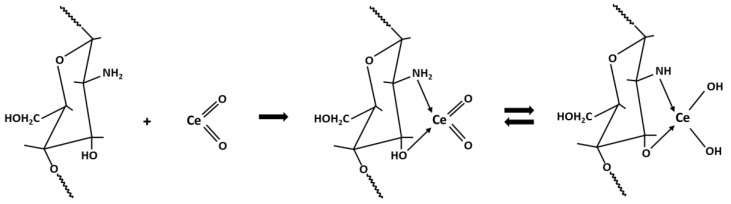
The formation of Ce-CTS composite hydrogel beads.

The hydrated cerium(IV) oxide ion is a yellow gelatinous precipitate, which is readily soluble in acids while being insoluble in excess base; it has a very well-developed surface and probably has good adsorption properties [36]. During the gelation process of the chitosan beads, cerium ions, in the presence of the hydroxyl groups introduced into the solution with NaOH, form the CeO_2_ hydrate while the reactive functional groups in chitosan, -NH_2_ and -OH, having free electron pairs, are coordinated by the Ce^4+^ ions [37]. In this study, the presence of peaks corresponding to the crystalline structure of CeO_2_ in resulting Ce-CTS was confirmed by XRD analysis. A similar result was obtained by Hu et al. [32] and Zhang et al. [38]. A monocomplex is probably formed between Ce and CTS (in a ratio of 1:1), as shown in Scheme 1, although a dichelate complex (1:2) could not be ruled out, as reported by Elanchezhiyan et al. [37]. The formation of a cerium mono or dichelate complex results in chitosan surface modification. The presence of additional OH groups promotes the chemical adsorption of boron.

### 2.3. pH Study and the Mechanism of B Adsorption

pH is an important parameter that should be considered when studying boron adsorption from aqueous solutions. This parameter determines the number of anionic species (B(OH)_4_^−^) and boric acid molecules (B(OH)_3_) present in the solutions that are in the equilibrium described by the equation below and the equilibrium constant K_a_:

B(OH)_3_ + H_2_O ↔ B(OH)_4_^−^ + H^+^   pK_a_ = 9.2 (3)

**Figure 4:**
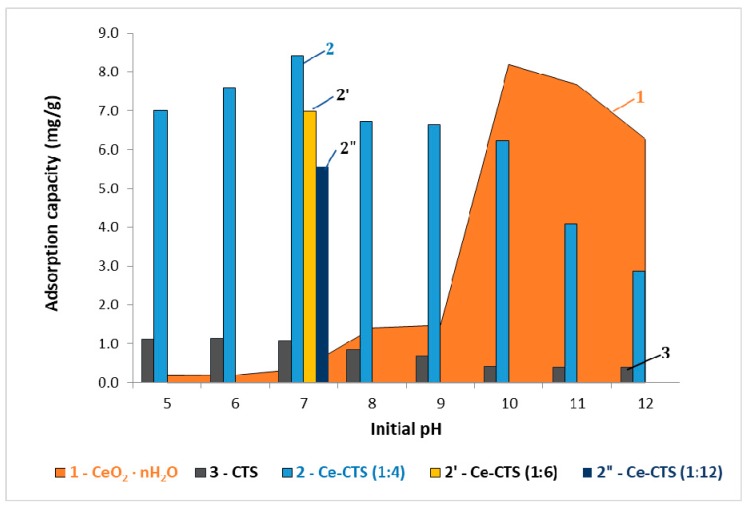
Comparison of boron adsorption capacity of CeO_2_·nH_2_O, Ce-CTS and CTS as a function of initial pH value at a temperature of 20 ± 1 °C.

Figure 4 shows how the adsorption of boron on freshly precipitated cerium(IV) oxide (graph 1), on chitosan modified with cerium(IV) (graph 2) and on unmodified CTS (graph 3) changed with the pH of the solution. When the pH increased from 5 to 7, boron removal by the Ce-CTS beads increased very slightly to reach the maximum adsorption of 8.42 ± 0.61 mg/g_(Ce)_ (calculated per 1 g of Ce) at pH 7.0. At a pH < 7, the adsorption of boron on CeO_2_·nH_2_O in situ was very low in contrast to the adsorption on CTS, which was the highest in low pHs. At a pH higher than 7, less boron adsorption on Ce-CTS was observed, while on CeO_2_·nH_2_O slowly increasing adsorption was noted (Figure 4). The maximum boron adsorption on hydrated cerium dioxide, at a value of q_expt_ = 8.20 ± 0.16 mg/g_(Ce)_, was reached at a pH of 10. The adsorption was only slightly decreased with a further increase in pH. On the other hand, when the pH was 10 and higher, rather low adsorption capacity of Ce-CTS was observed, which might have been due to the excess concentration of hydroxyl groups, which compete with borate anions in the alkaline solution [39]. The most beneficial adsorption effect was obtained at a pH of 10 and a pH of 7 for the process carried out on CeO_2_·nH_2_O and Ce-CTS, respectively. Furthermore, the adsorption on Ce-CTS depended on the content of Ce in the hydrogel. The capacity increased when the weight ratio of Ce to CTS increased from 1:12 to 1:4 (2, 2′ and 2” graphs in Figure 4). Adsorption on the unmodified CTS was very low and was only 1.1 mg/g at acidic pH (graph 3). This means a different adsorption mechanism occurs in these cases.

**Scheme 2:**
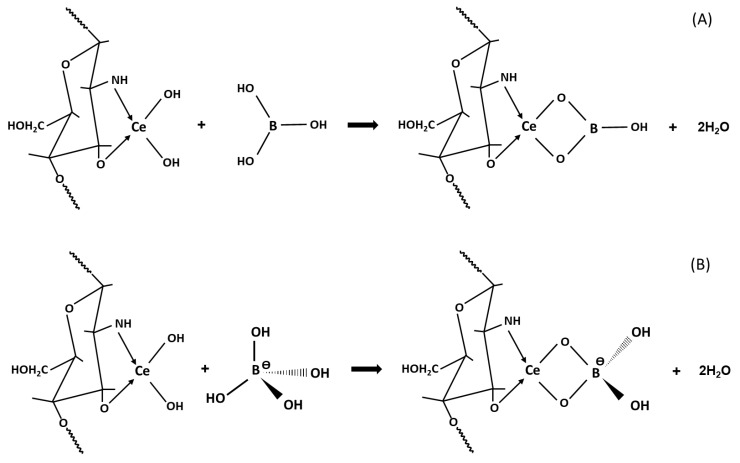
The possible mechanism of boron adsorption on the Ce-CTS composite hydrogel beads: (**A**) acid and neutral pH; (**B**) alkaline pH.

A mechanism of boron adsorption strongly depends on pH solution [39,40]. In the case of the unmodified CTS, when pH is below the pK_a_ value of chitosan (6.3–6.8), amine groups are protonated and act as the sites of formation of a complex with boric acid present in low pH. At a pH >6.8, adsorption on CTS occurs through the molecular attraction of H_3_BO_3_ through a hydrogen bond with an amine or hydroxyl groups of chitosan [40]. The adsorption of the B(OH)_4_^−^ anion on the hydrated CeO_2_·nH_2_O is presumably the result of electrostatic attraction between the positively charged surface of the oxide and the negative boron ion. In the case of adsorption on Ce-CTS, the adsorbent–adsorbate interaction can be explained as follows: because the maximum boron adsorption on Ce-CTS was observed at a pH of ca. 7 when the percentage of boric acid as a neutral monomolecule was more than 99% in the solution, it can be assumed that the Coulomb attraction was very weak or did not occur at all. The dominant mechanism of adsorption at neutral and acidic pH would be the reaction between the boric acid molecule and hydroxyl groups bound to the cerium chelated by chitosan active centres (Scheme 2A). At a pH >8 the amount of boron anionic species increases in the solutions, which results in the possibility of the reaction shown in Scheme 2B.

### 2.4. Isotherm Models

**Figure 5:**
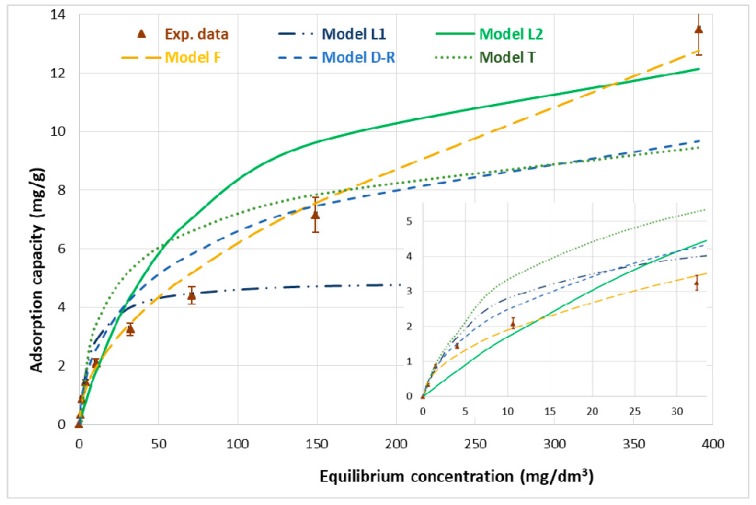
Boron adsorption isotherms on the Ce-CTS composite hydrogel beads: experimental curve and Langmuir, Freundlich, Dubinin–Radushkevich and Temkin models at 20 ± 1 °C and time of 24 h. The inserted figure is the adsorption isotherms on Ce-CTS with initial boron concentrations up to 50 mg/dm^3^.

Figure 5 shows the adsorption isotherm of boron on the Ce-CTS composite hydrogel beads and the fitting of the Langmuir, Freundlich, Dubinin–Radushkevich and Temkin adsorption models [41,42] onto the experimental data. The experimental adsorption capacity, *q*_expt_, was 13.5 ± 0.9 mg/g at 20 °C, with a contact time of 24 h and an initial boron concentration of 500 mg/dm^3^. Furthermore, the content of boron in the thermally dried Ce-CTS composite (after dissolving three samples in concentrated HNO_3_) was equal to 13.0 ± 0.3 mg/g, which confirmed the experimental adsorption capacity given above calculated according to Equation (11) (Section 3.4).

The adsorption capacity of the chitosan hydrogel beads towards boron was higher than that of the majority of chitosan adsorbents reported so far [3,15] or at least comparable to them [43,44]. The details are summarised in Table 1. Furthermore, it was found that Ce-CTS was a better biosorbent than the gelatinous precipitate of hydrated cerium dioxide. CeO_2_ had a boron adsorption capacity equal to 8.20 mg/g_(Ce)_, while Ce-CTS was 8.42 mg/g_(Ce)_ starting from the same initial boron concentration of 20 mg/dm^3^, as mentioned in Section 2.3. Additionally, the fine and difficult-to-separate precipitate of hydrated cerium dioxide has great disadvantages in water treatment technology, in contrast to the form of the hydrogel beads of the Ce-CTS composite proposed in this study. It should also be emphasised that boron is removed in high efficiency over a wide pH range, which makes Ce-CTS competitive with boron selective resins, e.g., Amberlite IRA 743, Purolite S 108 (see Table 1).

To assess the maximum adsorption capacity and to determine the mechanism of adsorption, two-parameter models of isotherms describing experimental data were used: the Langmuir, Freundlich, Dubinin–Radushkevich and Temkin equations [41,42]. The most developed equations used to describe adsorption equilibria are mainly the Langmuir and Freundlich models. The Langmuir (L) model assumes that adsorption occurs on surface sites on each of which the energy is equal, while the Freundlich (F) model allows for several kinds of adsorption sites in the solid, each having a different energy of adsorption. The Dubinin–Radushkevich (D–R) isotherm is a local isotherm often used to describe adsorption, explaining the equilibrium of adsorption by the theory of volume filling of adsorbent micropores. The Temkin (T) isotherm describes monolayer adsorption on a heterogeneous surface, assuming that the heat of adsorption of all the molecules in the layer decreases linearly due to the adsorbent–adsorbate interaction, and the adsorption is characterised by an even distribution of binding energy.

The results of the boron adsorption modelling are presented in Table 2. The adsorption models used in this study and their equations in linear and nonlinear forms are presented in the Supplementary Materials (Table S1). The coefficients of the models were determined by linear regression. The degree of fit of the linear equations to the experimental points was assessed on the basis of the coefficient of determination, *R*^2^:

(1) $$R^{2}=\frac{{\left(q_{expt}-{\bar{q}}_{calc}\right)}^{2}}{\sum {\left(q_{expt}-{\bar{q}}_{calc}\right)}^{2}+{\left(q_{expt}-{\bar{q}}_{calc}\right)}^{2}}$$

where *q_expt_* is an experimental adsorption capacity and *q_calc_* is a calculated adsorption capacity. The determined linear regression parameters were introduced into the model equations and the fit was rated using the mean error, *ARE*:

(5) $$ARE=\frac{100}{n}\overset{n}{\underset{i=1}{\sum }}\left|\frac{q_{expt}-q_{calc}}{q_{expt}}\right|$$

As presented in Table S1 and Table 2, *q_m_* and *B* are the L parameters, *q_m_* is the adsorptive capacity (mg/g) expressed as the maximum amount of boron that can be adsorbed by the adsorbent as a monolayer, and *B* is an equilibrium constant that corresponds to the adsorption energy (dm^3^/mg). The parameters *K*_F_ (mg/g) and *n* resulting from the F model correspond to the relative adsorptive capacity and the adsorption intensity of the adsorbent, respectively. The essential characteristics of a Langmuir isotherm can be expressed in terms of a dimensionless constant separation factor, *R_L_*, which is defined by Equation (6):

(6) $$R_{L}=\frac{1}{1+B\times C}$$

where C is final boron concentration in the solution (mg/dm^3^). According to Hall et al. [50], the parameter *R_L_* indicates the shape of the isotherm in the following manner: *R*_L_ > 1, unfavourable; *R*_L_ = 1, linear; 0 < *R*_L_ < 1, favourable; and *R*_L_ = 0, irreversible. Similarly, the fitness of using the F equation to describe the adsorption can be assessed by the constant *n*. If 1 < *n* <10, then the F equation is adequate for use [45].

In the D–R equation given in Table S1, *ε* is a Polanyi potential, which equals RTln(1+1/c), where *R* is a gas constant (kJ/(mol K)) and *T* is a temperature (K) while the *x*_m_ parameter is the adsorption capacity (mol/g) and the *k* parameter is a constant related to the adsorption energy (mol^2^/kJ^2^). The adsorption energy, E, (the energy required to transfer 1 mol of adsorbate species to the surface of the adsorbent from infinity in the bulk of the solution) is obtained from the following Equation (7):

(7) $$E=-{\left(2\times k\right)}^{-0.5\;}$$

The parameters b_T_ (kJ/mol) and A_T_ (dm^3^/mg), calculated from the T model, correspond to the adsorption energy change and the adsorption equilibrium constant, respectively. If the energy of adsorption is less than 20 kJ/mol, the adsorption is physical in nature due to weak van der Waals forces. The energy for chemisorption lies in the range 40–800 kJ/mol [45].

As can be seen in Table 2, the L model gave the best fit to the experimental data of boron adsorption on the Ce-CTS composite, because it had the highest correlation coefficient, 0.9897. The dimensionless constant separation factor, *R*_L_, in the range of <0.795 and >0.015 for initial boron concentrations of 2–500 mg/dm^3^, indicated a favourable adsorption. Unfortunately, the value of parameter *q*_m_, very different from the experimental capacity, *q*_exp_, indicated the impossibility of the full interpretation of the process by the Langmuir model. The smallest mean error, 13.2%, was obtained by fitting the F model simultaneously with a rather high correlation coefficient, *R*^2^ = 0.9810. As can be seen in Table 2, the *K*_F_ parameters amounted to 0.584 mg/g, while the parameter *n* was 1.95, which meant that the F model was adequate for describing the discussed process. A slightly worse fit was obtained for the D–R model: the correlation coefficient was 0.9097, while the mean error was 26.7%. Although the D–R model was less suitable than the F model, the calculated value *X*_m_ (14.4 mg/g) was in a good agreement with the experimental *q*_expt_, 13.5 ± 0.9 mg/g. The worst fit was obtained for the T model, which was inadequate for describing boron adsorption on Ce-CTS. 

Generally, the Freundlich equation fits the experimental data because of its high correlation coefficients and relatively low mean error. The better conformity of the Freundlich model over the other isotherm models for boron adsorption was noted on freeze-dried chitosan beads [26], freeze-dried chitosan functionalised with N-methylglucamine [46] and cobalt(II)-doped chitosan hydrogel [51]. The F model usually corresponds to multilayer adsorption with non-uniform distribution of adsorption heat and affinities over the heterogeneous surface. The values of parameters, *k_f_* below 1 and *n* above 1, indicate high adsorption intensity and the chemisorption nature of the process [52]. 

### 2.5. Kinetic Models

**Figure 6:**
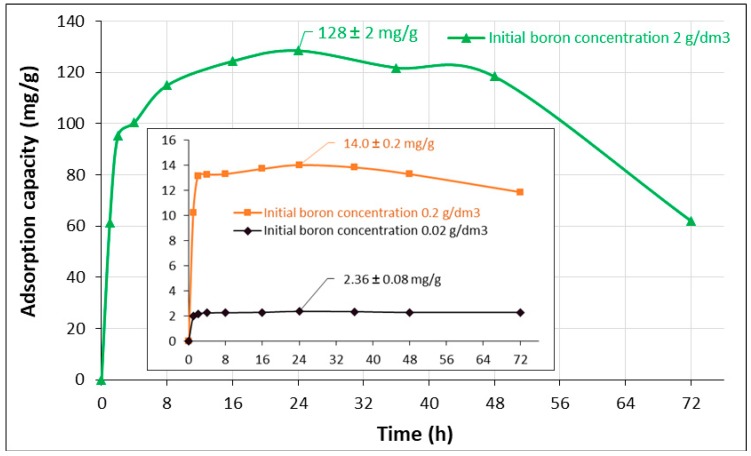
Effect of contact time on boron adsorption efficiency using the Ce-CTS composite hydrogel beads. The inserted figure is the effect of contact time with initial boron concentrations of 20 and 200 mg/dm^3^.

The effect of contact time, *t*, on boron adsorption is shown in Figure 6. Equilibrium was reached after 24 h, independently of the initial boron concentration. Four kinetic models: the Lagergren pseudo-first-order, the Elovich pseudo-second-order, the parabolic diffusion and the combination of both the chemical reaction and the intraparticle diffusion models were tested [53,54,55]. The kinetic model equations in linear and nonlinear forms are presented in the Supplementary Materials (Table S2). The coefficients of the models were determined by linear regression.

Moreover, the constant *k*_2_ was used to calculate the initial adsorption rate, *r*, at *t* = 0, defined by Equation (8):

(8) $$r=\;k_{2}\times {\left(q_{e}\right)}^{2}$$

In Table 3, the resulting parameters and the correlation coefficients *R*^2^ of the kinetic models for various initial boron concentrations are listed. The calculated values of *R*^2^ for the pseudo-first-order and the intraparticle diffusion kinetic equations were low, indicating that the Elovich and Weber and Morris models were not applicable to the discussed process. However, in the high initial boron concentration, good agreement was obtained when the model of pseudo-first order reaction was used. The description by the pseudo-second-order reaction model gave very good correlations with the experimental data. For the pseudo-second-order rate kinetic model, the coefficients *R*^2^ were in the range of >0.9995 and <0.9997. The calculated values of *q*_e_ agreed with the experimental adsorption capacities, *q*_expt_, while the initial adsorption rate, *r,* increased when the initial boron concentration increased. Unfortunately, the chemical rate constants for the second-order reaction were the function of initial concentrations of the boron solution—they decreased 237 times with an increase of the concentration from 20 to 2000 mg/dm^3^. Hence, on the basis of the reaction models, it was difficult to draw a clear conclusion on the mechanism that governed the adsorption rate. To describe this process, a model combining the chemical reaction with intraparticle diffusion was proposed [55]. The results indicate that the combined model was more appropriate to describe boron adsorption on the solid surface of the Ce-CTS composite. A parameter *k_4_* called the surface process constant by Modrzejewska et al. [55] was used to describe sorption of copper by chitosan hydrogel; it was independent of the concentration. In Table 3, the resulting rate constants for the combined model insignificantly decreased with an increase in the concentration from 20 to 2000 mg/dm^3^. Hence, it was assumed that the process of diffusion inside the granule structure was proceeded by irreversible adsorption on the inner granule walls.

### 2.6. The Stability of the Ce-CTS and Desorption Tests

The stability of the composite, and, in particular, the bond strength between cerium and chitosan, is an important factor for qualifying the proposed biosorbent for use in purifying water or wastewater. In this study, the degree of elution of cerium from the composite was tested under the conditions of the adsorption and desorption process. 

**Figure 7:**
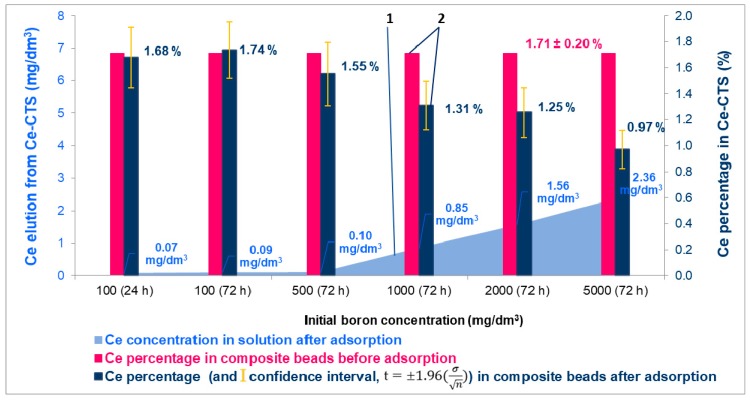
The influence of the initial boron concentration and time of contact on the elution of cerium ions from the Ce-CTS composite hydrogel beads (1) and changes of the cerium percentage in Ce-CTS during adsorption (2).

The effect of contact time and initial boron concentration on the elution of cerium ions from the Ce-CTS composite beads during the adsorption at a pH of 7 is shown in Figure 7. The graph shows that Ce(IV) ions did not elute from the composite, even after 3 days of contact time with the adsorptive, when the initial concentration of boron was up to 500 mg/dm^3^. The percentage of the cerium in the Ce-CTS before and after adsorption indicated that there were no changes in the content of cerium in the biosorbent. However, when the initial concentration of boron in solution increased, the elution of cerium ions also slightly increased. The elution of the cerium(IV) in the concentration range from 0.5 to 2.5 mg/dm^3^ was noticeable only when the initial boron concentration was 1000 mg/dm^3^ or greater, and when the contact time was elongated to 3 days. As can be seen in Figure 7, the increase in the cerium concentration in the eluate was accompanied by a reduction in the cerium content in the composite, which varied from 1.55 to 0.97, while the boron concentration increased from 500 to 5000 mg/dm^3^ and the contact time was 72 h. Because the optimal adsorption time was set to 24 h (see Figure 6), and after this time no leaching of the cerium from the composite was found, it was concluded that the proposed biosorbent was stable under the conditions of the adsorption process.

**Figure 8:**
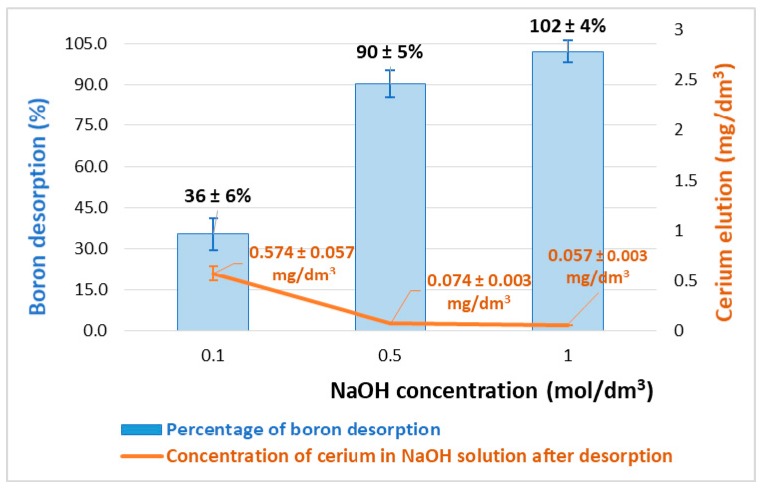
The influence of the regenerant concentration on boron desorption efficiency and cerium elution from the Ce-CTS composite hydrogel beads.

Next, the possibility of desorbing the boron from the composite without the loss of cerium in the composite was considered. The percentage of boron desorption, *D* (%), was calculated from the experimental data using the following equation:

(9) $$D=\frac{q_{D}}{q_{expt}}\times 100$$

where *q*_D_ is the amount of desorbed boron per biosorbent mass (mg/g). Examining the effect of pH (Figure 4), the adsorption capacity of B(III) on Ce-CTS was relatively low under alkaline conditions. Furthermore, it is known that CeO_2_ is insoluble in excess base. Therefore, the B desorption process was carried out using various concentrations of sodium hydroxide solution. The effect of the regenerant concentration on boron desorption and on cerium elution can be seen in Figure 8. Total boron desorption was obtained with 1 M NaOH solution. No Ce was found in the regenerative solution after desorption. The obtained results indicate that the Ce-CTS composite can be effectively regenerated and reused in the next adsorption cycle.

## 3. Materials and Methods 

### 3.1. Reagents

All the reagents employed in the study were of analytical reagent grade. A basic standard solution of boron in the form of boric acid (1 g/dm^3^) and cerium standard solutions of 1 g/dm^3^ were supplied by Merck. Water was purified by a Millipore Elix 10 system. Chitosan (molecular weight 600,000–800,000) was purchased from Acros Organics (Geel, Belgium); its degree of deacetylation, as determined by means of an H^1^ NMR method, was equal to 97%. Cerium nitrate, Ce(NO_3_)_4_·6H_2_O, was supplied by Loba Feinchemie GmbH (Fischamend, Austria). Sodium hydroxide, hydrochloric acid and glacial acetic acid were purchased from Avantor Performance Materials Poland S.A., Gliwice, Poland.

### 3.2. Analytical Procedures and Apparatus 

Boron and cerium were determined using inductively coupled plasma optical emission spectroscopy (ICP-OES) with a Varian 710-ES spectrometer (Varian, Mulgrave, Victoria, Australia). The following parameters were used: radio frequency (RF) power 1.0 kW, plasma flow 15 dm^3^/min, auxiliary flow 1.5 dm^3^/min, nebuliser pressure 200 kPa, pump rate 15 rpm and emission lines: λ = 208.956, λ = 249.678 and λ = 249.772 nm (for boron) and λ = 407.347, λ = 407.570, λ = 418.659 and λ = 446.021 nm (for cerium). Calibration curves were prepared using standard solutions in the ranges of 0.1–1.5 mg/dm^3^ and 0.1–5 mg/dm^3^ for boron and cerium, respectively. The changes in the chitosan structure were determined by a Fourier-transform infrared (FTIR) Spectrum Two spectrometer (Perkin Elmer, Waltham, MA, USA). The scanning electron microscope (SEM) micrographs of the chitosan bio-composite were produced using a Phenom ProX SEM (Phenom-World Bv, Eindhoven, The Netherlands). The phase compositions of the chitosan-based samples were determined using a Seifert 3003TT powder X-ray diffractometer with a Cu X-ray tube: kλ_1_ = 1.540598 Å, kλ_2_ = 1.544426 Å and kβ = 1.39225 Å (Seifert, Ahrensburg, Germany). Powder samples were analysed three times, and the XRD patterns presented in the manuscript were an average of the scans between 10 and 90° 2Theta.

### 3.3. Ce-CTS Composite Preparation

The procedure described previously [46] was adopted to prepare cerium–chitosan hydrogel beads. Briefly, a chitosan solution was prepared by dissolving 3 g of chitosan in 75 cm^3^ of 1 wt.% acetic acid solution. Next, 2.65 g, 1.56 g or 0.76 g of Ce(NO_3_)_4_·6H_2_O (the precursor of the oxide/hydroxide CeO_2_/Ce(OH)_4_) was added to the chitosan solution and sonicated for 30 min to obtain a well-dispersed, homogenous solution. The chitosan-precursor solution was added dropwise, using a thin needle (internal diameter 0.9 mm), into a stirred 20 wt.% aqueous NaOH solution. This resulted in immediate coagulation and the formation of beads. The newly formed chitosan beads (with different weight ratios of Ce to CTS: 1:4; 1:6 and 1:12) were then filtered and washed with demineralised water to remove traces of gelling solution. A portion of the chitosan beads was dried at 70 °С for 12 h to remove the water from the pore structure. The total content of cerium in the composite was determined by the ICP-OES method after dissolving the thermally dried chitosan beads in concentrated HNO_3_. The dry beads were powdered in an agate mortar and sieved to a constant size of <0.45 mm. The averaged Ce-CTS powder (weight ratio of Ce to CTS equal to 1:4) was analysed by FTIR and XRD.

### 3.4. Adsorption and Desorption Procedure

The experiments were carried out with 1 g of composite hydrogel beads and 0.01 or 0.02 dm^3^ of boron solution at a concentration range of 0–5000 mg/dm^3^. The experiments were carried out at a pH range of 5–10 and a temperature of 20 °C for 1–72 h in a 100 cm^3^ conical flask with a ground polyurethane (PU) joint. The boron solution and composite hydrogel beads were shaken at a mixing rate of 60 rpm in a mechanical shaker. At the end of the experiment, the residual solution was analysed for boron and cerium concentration by the ICP-OES method. Each adsorption experiment was repeated three times to obtain an average value. The content of adsorbed boron was also determined by the ICP-OES method after dissolving the composite samples in concentrated HNO_3_.

The boron adsorption coefficients (the removal efficiency, *R* (%), and the adsorption capacity, *q*_expt_ (mg/g)) were calculated from the experimental data in each sample using the following equations:

(10) $$R=\frac{\left(C_{0}-C\right)}{C_{0}}\times 100$$

(11) $$q_{expt}=\frac{\left(C_{0}-C\right)}{m}\times V_{0}$$

where *C*_0_ and *C* are the initial and final concentrations of boron in the solution (mg/dm^3^), respectively, *V*_0_ is the volume of adsorptive (dm^3^) and *m* is the dry mass of the composite or the equivalent mass of cerium (g).

The desorption of boron from the composite was examined as follows: 1.0 g of boron-saturated composite hydrogel beads and 20 cm^3^ of sodium hydroxide solution at concentrations of 0.1, 0.5 or 1.0 mol/dm^3^ were mixed for 24 h at room temperature. The boron and cerium concentrations were determined by taking aliquots. The concentrations given were the means of three experimental results.

The behaviour of boron and cerium species present together in the solution at increasing pH was examined. For this purpose, a solution containing Ce(IV) at a concentration of 2000 mg/dm^3^ and B(III) at a concentration of 20 mg/dm^3^ was made. The pH of the solution was adjusted to a value of 2 by using 0.1 M HCl. Then, 0.4 cm^3^ of the solution was used for the analysis of boron and cerium by the ICP-OES method. Next, 0.1 or 2 M NaOH solution was added to increase the pH by one unit, and 0.4 cm^3^ of the solution was again taken for the analysis of the boron and cerium concentration. The procedure was continued until the pH reached a value of 12.

## 4. Conclusions

A low-cost, non-toxic, biodegradable Ce-CTS composite hydrogel was prepared by an encapsulation technique for the efficient removal of B(III) from aqueous solutions. Ce-CTS possesses higher adsorption capacity than cerium dioxide hydrate (CeO_2_·nH_2_O) and chitosan hydrogel beads, the precursors of the new biosorbent. It was stable under the conditions of the adsorption–desorption processes. Its high adsorption capacity and convenient form, beads, guarantees its efficiency and ease of application in water purification. 

Boron removal by the Ce-CTS biosorbent was dependent on the pH of the solution, being most favourable at a pH of 7. The equilibrium data for boron adsorption on Ce-CTS fitted the Freundlich isotherm model, which indicated that the process was non-homogeneous and chemical in nature. The maximum adsorption was achieved after 24 h and followed the kinetic model combining the chemical reaction with intraparticle diffusion. 

The functionalisation of chitosan with Ce(IV) to improve the adsorptive behaviour of hydrogel beads was observed. SEM-EDS, FTIR, XRD and ICP-OES analyses confirmed the formation of a chelate complex between the Ce^4+^ ion and active groups on the chitosan surface. The dominant mechanism of removal was the reaction between the boric acid molecule or borate anion and hydroxyl groups bound to the cerium chelated by chitosan active centres. Considering the abovementioned factors, the proposed Ce-CTS composite beads could be a potential alternative for the effective removal of boron(III) from contaminated water.

## Figures and Tables

**Table 1:** Comparison of boron adsorption capacity on Ce-CTS with chitosan beads and other sorbents.

Sorbent/Parameter	Temp.(C)	pH	C_0_(B) (mg/dm^3^)	Adsorbent Dosage (g/dm^3^)	t (min)	q_expt_ (mg/g)	Ref.
Filler	**Chitosan**						
NanoTiO_2_	25	4	20	50	5	4.3	[15]
NanoCr_2_O_3_	25	4	20	50	5	3.5	[15]
NanoFe_3_O_4_	25	4	20	50	5	4.4	[15]
Fe(OH)_3_	25	4	20	50	5	7.8	[15]
Without filler	25	8	10	2	30	3.5	[26]
Glycidol	25	7	5.2	8.3	45	23.8	[39]
Ni(OH)_2_	25	7	50	1	4320	61.4 ***	[40]
NMDG *	20	7	4.8	0.5	480	35.1	[45]
Co(OH)_2_	25	8.5	20	50	60	2.5	[46]
Ce(OH)_4_	20	7	500	100	2880	13.5	This paper
	**Other sorbents**						
Amberlite IRA-743	30	9.5	40	10		7.5	[3]
AC ** and Salicylic Acid	25	8	25	20	30	0.325	[8]
AC ** and Curcumin	25	5.5	1000	40	120	5.05*	[9]
Fly ash zeolite	25	7	50	20	30	2.3	[12]
Cotton cellulose	25	7	500	20	480	11.3	[47]
Clinoptylolite	25	8	50	20	30	0.155	[48]
Clinoptylolite and ZrO_2_	25	8	50	20	30	1.03	[48]
Purolite S 108	30	9.2	****	2	****	6.2	[49]

*: N-methylo-D-glucamine; **: Activated carbon; ***: Calculated per 1 g of Ni(OH)_2_; ****: no data.

**Table 2:** Parameters of Langmuir (L), Freundlich (F), Dubinin–Radushkevich (D–R) and Temkin (T) isotherms on the Ce-CTS composite hydrogel beads at 20 ± 1 °C.

Isotherm Model	Parameters of the Isotherm Models (*q*_expt_ = 13.5 ± 0.9 mg/g)							
**L**	*q*_m_ (mg/g)		*B* (dm^3^/mg)		*R* ^2^		*ARE* (%)	
	4.95		0.129		0.9897		23.9	
	R_L_ for initial boron concentration (mg/dm^3^):							
	2	5	10	20	50	100	200	500
	0.795	0.608	0.437	0.279	0.134	0.072	0.037	0.015
**F**	*K*_F_ (mg/g)		*n*		*R* ^2^		*ARE* (%)	
	0.584		1.95		0.9810		13.2	
**D–R**	*X*_m_ (mg/g)		*E* (kJ/mol)		*R* ^2^		*ARE* (%)	
	14.4		−9.13		0.9709		26.7	
**T**	*A*_T_		*b*_T_ × 10^−3^		*R* ^2^		*ARE* (%)	
	0.727		1.48		0.7651		41.0	

**Table 3:** Kinetic parameters for boron adsorption on the Ce-CTS composite hydrogel beads at 20 ± 1 °C.

Initial Boron Concentration	Experimental Capacity	Parameters of the Kinetic Models			
		Pseudo-first-order			
C_0_(B) (mg/dm^3^)	q_expt_ (mg/g)	q_1_ (mg/g)	K_1_ (g/(mg∙h))	R_1_^2^	
20	2.36	0.148	−0.0368	0.1234	
200	14.0	1.026	0.0394	0.1927	
2000	128.5	58.425	0.1697	0.9864	
		Pseudo-second-order			
C_0_(B) (mg/dm^3^)	q_expt_ (mg/g)	q_2_ (mg/g)	k_2_ (g/(mg∙h))	R_2_^2^	r (mg/(g∙h))
20	2.36	2.37	1.540	0.9996	8.65
200	14.0	14.1	0.2167	0.9997	43.1
2000	128.5	133.3	0.0065	0.9995	114.9
		Intraparticle diffusion model			
C_0_(B) (mg/dm^3^)	q_expt_ (mg/g)	q_3_ (mg/g)	K_p_ (mg/(g∙(h)^1/2^))	R_3_^2^	
20	2.36	2.01	0.076	0.7273	
200	14.0	11.2	0.650	0.5269	
2000	128.5	65.0	14.5	0.8018	
	Model combining the chemical reaction with intraparticle diffusion				
C_0_(B) (mg/dm^3^)	q_expt_ (mg/g)	q_4_ (mg/g)	K_4_ (dm^3^/(g∙h))	R_4_^2^	
20	2.36	2.95	0.0099	0.9287	
200	14.0	103.5	0.0060	0.9081	
2000	128.5	1577	0.0034	0.8925	

## Supplementary Materials

Supplementary materials can be found at https://www.mdpi.com/1422-0067/20/7/1567/s1.

## Abbreviations

CTS	Unmodified chitosan
Ce-CTS	Chitosan composite supported with Ce(IV)
Ce-CTS-B	Chitosan composite supported with Ce(IV) after boron adsorption
FTIR	Fourier-transform infrared spectrometer
SEM-EDS	Scanning electron microscope with energy dispersive spectrometer
XRD	X-ray diffractometer
ICP-OES	Inductively coupled plasma optical emission spectrometer
L model	Langmuir model
F model	Freundlich model
T model	Temkin model
