# Supplementary material for: Chitosan Hydrogel Beads Supported with Ceria for Boron Removal
Source: Int J Mol Sci. 2019 Mar 28;20(7):1567. doi: 10.3390/ijms20071567 (PMC6479674; doi:10.3390/ijms20071567)
Supplement: Supplementary file 1 [file ijms-20-01567-s001.pdf]

Supplementary materials

Chitosan hydrogel beads supported with ceria for boron removal by Kluczka J. et al.

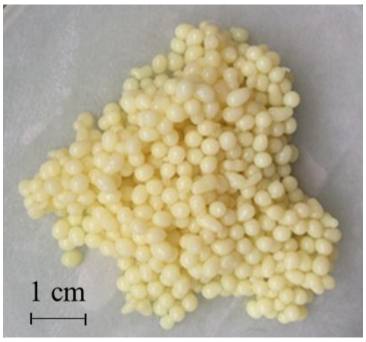

Figure S1 Photograph of Ce-CTS composite hydrogel beads.

Table S1 Langmuir, Freundlich, Dubinin-Radushkevich and Temkin models of adsorption isotherms.

Model	Nonlinear equation	Linear equation	Plot
L	$q_e = q_m \times \frac{B \times C_e}{1 + B \times C_e}$	$\frac{1}{q_e} = \frac{1}{q_m \times B \times C_e} + \frac{1}{q_m}$	$\frac{1}{q_e} vs \frac{1}{C_e}$
F	$q_e = K_F \times (C_e)^{1/n}$	$log(q_e) = log(k_F) + \frac{1}{n} log(C_e)$	$log(q_e)\ vs\ log(C_e)$
D–R	$q_e = (X_m)\ exp(-k \times \varepsilon^2)$	$ln(q_e) = ln(X_m) - k \times \varepsilon^2$	$ln(q_e)\ vs\ \varepsilon^2$
T	$q_e = \frac{R \times T}{b_T} ln\ (A_T \times C_e)$	$q_e = \frac{R \times T}{b_T} ln\ (A_T) + \frac{R \times T}{b_T} ln\ (C_e)$	$q_e\ vs\ ln(C_e)$

where: q_m (mg/g) and B (dm^3/mg) are the Langmuir parameters, K_F (mg/g) and n are the parameters resulting from the Freundlich model, ε , Polanyi potential, x_m (mol/g) and the k (mol^2/kJ^2) are the Dubinin-Radushkevich parameters, the parameters b_T (kJ/mol) and A_T (dm^3/mg) are calculated from the T model.

Table S2 Lagergren pseudo-first-order, Elovich pseudo-second-order, and Weber and Morris parabolic diffusion models of kinetics.

Kinetic model	Differential/Nonlinear equation	Linear equation	Plot
Pseudo-first-order	$\frac{dq_e}{dt} = k_1(q_e - q_t)$	$\log(q_e - q_t) = \log(q_e) - \frac{k_1 \times t}{2.303}$	$log(q_e - q_t)\ vs\ t$
Pseudo-second-order	$\frac{dq_e}{dt} = k_2(q_e - q_t)^2$	$\frac{t}{q_t} = \frac{1}{k_2 \times (q_e)^2} + \frac{t}{q_e}$	$\frac{t}{q_t}\ vs\ t$
Parabolic diffusion	$q_t = k_p \times t^{1/2}$	-	$q_t\ vs\ t^{1/2}$

where q_t and q_e are the amounts of boron adsorbed (mg/g) at any time t and at the time of equilibrium while k_1, k_2, and k_p are the pseudo-first-order, pseudo-second-order, and the parabolic diffusion rate constants, respectively.
